# Design and Evaluation of a Faculty Development Workshop Series on Integrating Generative Artificial Intelligence in Medical Education: Mixed Methods Pilot Study

**DOI:** 10.2196/89815

**Published:** 2026-05-06

**Authors:** Rajalakshmi Anand, Nicole Bowers, Mange Festo Manyama

**Affiliations:** 1 Institute for Population Health, Weill Cornell Medical College-Qatar, Qatar Foundation—Education City, Doha, Baladīyat ad Dawḩah, 24144, Qatar, 974 44928389; 2 Mary Lou Fulton College for Teaching and Learning Innovation, Arizona State University, Tempe, AZ, United States; 3 Division of Medical Education, Weill Cornell Medical College-Qatar, Doha, Qatar

**Keywords:** generative artificial intelligence, medical education, faculty development, experiential learning, TPACK, ethical AI, responsible AI

## Abstract

**Background:**

Generative artificial intelligence (GenAI) tools are being increasingly applied to teaching and learning in medical education creating both instructional opportunities and pedagogical challenges. While GenAI offers potential to enhance teaching, assessment, and curriculum design, many medical faculty lack structured guidance on how to integrate these tools ethically and pedagogically within discipline-specific, high-stakes educational contexts.

**Objective:**

This study aimed to design, implement, and evaluate a faculty development workshop series for ethical and pedagogical integration of GenAI in medical education teaching.

**Methods:**

A mixed methods pilot study was conducted to design, implement, and evaluate a faculty development workshop series “Professional Development in Generative Artificial Intelligence for Pedagogy” at Weill Cornell Medicine-Qatar, a US medical school in Qatar. The program consisted of five 1-hour synchronous online workshops grounded in Experiential Learning Theory and the Technological Pedagogical Content Knowledge framework. Ten medical faculty from multiple disciplines participated. Quantitative data were collected through an online preintervention survey, an online postintervention survey with open-ended questions, and an online 2-week follow-up survey. Surveys consisted of 5-point Likert scale items capturing perceptions of workshop quality, confidence, and intended application. Qualitative data included full workshop transcripts, facilitator theoretical notes, and facilitator memos. Descriptive statistics summarized quantitative findings, while qualitative data were analyzed using a combination of deductive and inductive coding, alongside narrative analysis. Findings were integrated to generate convergent interpretations.

**Results:**

Qualitative analysis of workshop transcripts suggested evolving engagement with GenAI, with participants describing movement from exploratory use toward more intentional pedagogical application. Postintervention survey results indicated high satisfaction with program content, organization, relevance, and overall quality. Two-week follow-up survey responses (n=5) suggested increased self-reported confidence in applying GenAI tools, and perceived shifts in how participants conceptualized teaching with GenAI. Faculty described intended strategies for integrating GenAI into lesson planning, assessment design, visualization of learning materials, and case-based instruction, while emphasizing the importance of human oversight, critical appraisal, and ethical judgment. Findings highlighted the perceived value of hands-on experimentation, reflective discussion, and adaptive facilitation in supporting early faculty engagement.

**Conclusions:**

This pilot study provides early evidence that an experiential, theory-informed, and adaptively facilitated faculty development workshop series may support medical faculty in developing self-reported confidence, awareness, and initial strategies for responsible GenAI integration. Findings are exploratory and limited by a small sample size, a single institution, and reliance on self-reported data. Nevertheless, the Professional Development in Generative Artificial Intelligence for Pedagogy workshop series presents a flexible and theory-informed faculty development approach that may inform future faculty development initiatives in medical education as GenAI technologies continue to evolve.

## Introduction

### Background

The emergence of generative artificial intelligence (GenAI) tools such as ChatGPT in November 2022 sparked widespread concern and curiosity among medical faculty, many of whom at that time lacked clarity on the capabilities, limitations, and ethical implications of the use of GenAI in medical education (ME) pedagogy. ChatGPT became one of the fastest growing consumer applications in history [1,2]. Unlike static instructional technologies, GenAI tools are protean, generative, opaque, unstable, and context-sensitive [3]. While these characteristics enable novel forms of instructional support, they also introduce uncertainty, particularly when GenAI tools are adopted faster than educators can develop responsible and theory-informed faculty development approaches for their use [4].

At Weill Cornell Medicine-Qatar, a US medical school in Doha, Qatar, the rapid emergence of GenAI technologies highlighted the need to proactively support educators in navigating this emerging landscape [5]. This institutional experience reflected a broader concern of faculty in academic medicine during 2023‐2024. Although research from related fields highlights the value of ongoing professional development (PD) for building GenAI competence [1], existing PD approaches do not adequately meet the needs of experienced medical educators working under intense time constraints. ME educators are disciplinary experts whose instructional decisions carry professional accountability and implications for patient care [5]. Without structured, contextually relevant learning experiences, GenAI may risk being adopted primarily for efficiency, with limited attention to pedagogical alignment or ethical considerations [6,7]. Inaccuracies, oversimplifications, or uncritical reliance on automated GenAI outputs may shape learners’ clinical reasoning and professional formation [8,9]. Additionally, reliance on automated GenAI outputs also raises the risk of cognitive deskilling, whereby higher-order pedagogical reasoning and instructional decision-making may be diminished through overautomation rather than being exercised and refined [10,11].

While studies have demonstrated growing interest in GenAI’s potential, few have empirically examined its ethical and pedagogical use within ME settings [9,12,13]. Emerging scholarship has raised concerns that frequent delegation of complex cognitive tasks to GenAI systems may shift educators from active decision-making toward uncritical or nonpurposeful reliance on generated content, with implications for teaching quality and professional expertise [14-16]. These risks underscore the need for faculty development approaches that explicitly position GenAI as a tool for critique and reflection rather than a substitute for pedagogical reasoning. These risks are amplified for experienced educators who are pressed for time and navigating rapidly evolving technologies without structured opportunities for critical examination.

A growing body of ME scholarship has begun to describe institutional readiness and emerging approaches to GenAI training and guidance for faculty and learners. For example, recent work has reported variability in GenAI policy and training availability across ME institutions and highlighted the need for structured support for faculty implementation [17,18]. Other studies have described targeted faculty development workshops aimed at building practical GenAI literacy and teaching-oriented application, underscoring both interest and variability in preparedness [19,20]. This emerging evidence base supports the need for context-specific faculty development interventions that move beyond general awareness toward educator-directed and pedagogically aligned use [21-23]. Unlike many other educational contexts, ME is embedded within frameworks of professional accountability and ethical responsibility. As a result, integrating GenAI into teaching requires approaches that preserve the central role of human expertise and judgment. Faculty development initiatives must therefore move beyond tool familiarization to support reflective, context-sensitive engagement with GenAI outputs, positioning these tools as assistive resources rather than substitutes for pedagogical reasoning.

Guided by these considerations, we designed the Professional Development in Generative Artificial Intelligence for Pedagogy (PDGenAI-P) workshop series as a faculty development intervention for medical educators [5]. Grounded in the Technological Pedagogical Content Knowledge (TPACK) [3,24] framework and Experiential Learning Theory (ELT) [25,26], PDGenAI-P was structured to support hands-on experimentation with GenAI tools alongside guided critique and structured reflection on pedagogical alignment and ethical considerations. This study did not evaluate the effectiveness of GenAI tools, nor did it compare instructional modalities or measure changes in teaching performance. Instead, it describes the design, implementation, and pilot evaluation of a theory-informed PD approach that supported a small medical faculty cohort (n=10) in critically engaging with GenAI within their pedagogical contexts. By centering disciplinary expertise, reflective judgment, and ethical awareness, PDGenAI-P offers an exploratory contribution to emerging scholarship on faculty development for GenAI integration in ME.

### Program Design

PDGenAI-P [5] was designed to address a gap in context-specific PD that recognizes medical educators’ disciplinary expertise while supporting pedagogical and ethical engagement with GenAI. The program consisted of 5 live, online 1-hour workshops delivered via Zoom (Zoom Communications, Inc). Two scheduling formats were offered to accommodate faculty schedules: (1) a 5-day format with 1-hour workshops or sessions each day (July 2024), and (2) a condensed 2-day format with multiple workshops or sessions per day (August 2024). Each workshop session was approximately 1 hour in duration in the 5-day format; in the condensed format, multiple 1-hour sessions were delivered across 2 days. Total instructional time across the 5 workshops was approximately 5 hours. Each workshop built upon the previous one, moving from foundational exploration of GenAI tools toward context-specific pedagogical application.

The program’s learning objectives were to support medical educators to (1) analyze key characteristics, capabilities, and limitations of GenAI within ME contexts; (2) evaluate transparent and responsible GenAI practices that foreground human expertise and educator decision-making; (3) design pedagogically sound learning activities by integrating GenAI with medical educators’ disciplinary and instructional expertise; (4) evaluate ethical and pedagogical challenges associated with GenAI use in ME teaching and exercise professional judgment in mitigating them; and (5) value human expertise, confidence, and professional judgment when engaging with GenAI as an assistive, rather than substitutive, pedagogical resource.

These learning objectives guided the overall program design and facilitation but were not explicitly revisited as assessment criteria during workshops. The PDGenAI-P workshop series was developed as part of a doctoral dissertation and represents the second iteration of an evolving faculty development initiative [5]. The initial iteration was delivered in a fully self-paced format. Between October 2023 and April 2024, the program design was iteratively refined through informal general and targeted needs assessment, including review of feedback from the first self-paced iteration, consultation with faculty colleagues, and reflection on emerging pedagogical and ethical concerns related to GenAI use in ME. The 5-workshop structure and pedagogical focus areas were finalized in consultation with various medical, GenAI, and other faculty experts by April 2024 to support institutional review board approval. Following delivery of the first live iteration in July 2024, the program was further reviewed and modestly adapted based on participant feedback prior to the subsequent August iteration. This iterative design process was intended to ensure that the program remained contextually relevant, needs-sensitive, and responsive to medical educators’ evolving concerns regarding GenAI integration.

**Figure 1.**
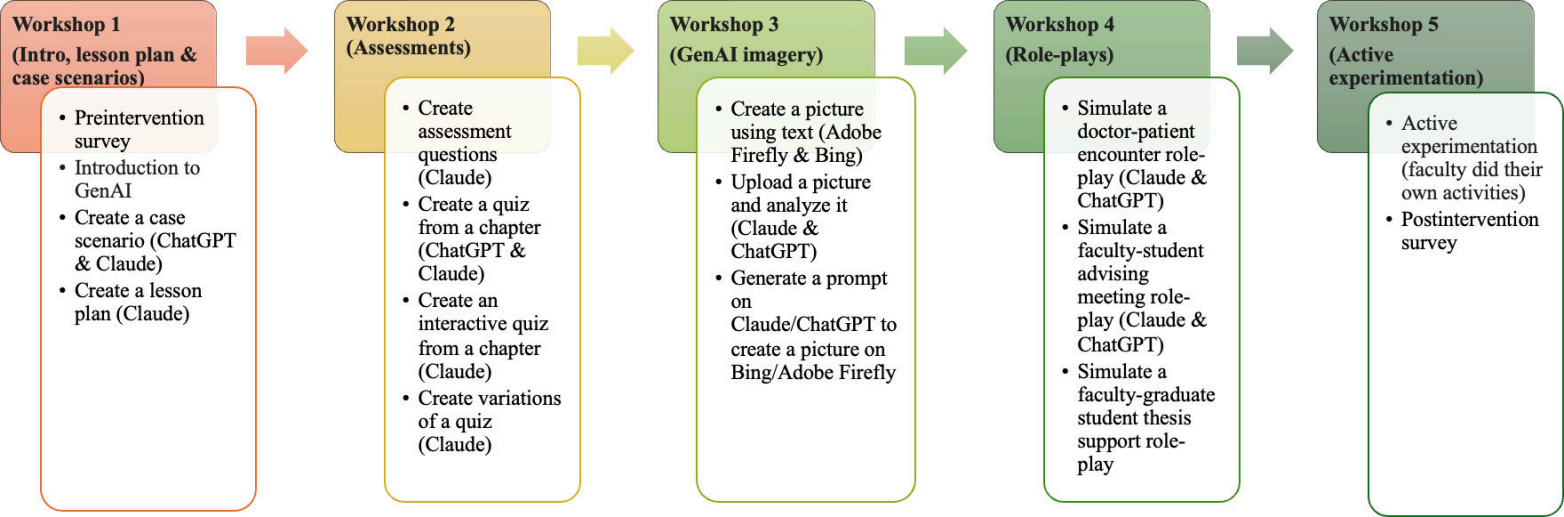
Overview and content focus of the Professional Development in Generative Artificial Intelligence for Pedagogy workshops.

An overview of the PDGenAI-P workshops and their pedagogical content focus is shown in Figure 1. Workshops focused on lesson planning and case scenario design (workshop 1), assessments (workshop 2), GenAI imagery (workshop 3), role-play development (workshop 4), and independent experimentation (workshop 5). Learning activities were fully scaffolded through structured prompts provided by the facilitator, with limited flexibility intentionally built in to allow participants to adapt outputs to their own teaching contexts. Activities followed an experiential learning cycle, beginning with facilitator-led demonstration, followed by individual experimentation, and culminating in structured group reflection and abstract conceptualization.

An overview of each workshop’s focus and examples of scaffolded GenAI prompts used during activities is available in Multimedia Appendix 1. During workshops, participants generated tangible pedagogical artifacts, such as draft lesson plans, assessment items, role-play scripts, and image-based materials. While activity worksheets were provided as optional scaffolds to guide prompt use and documentation, artifacts were not collected, graded, or formally analyzed. To mitigate risks of cognitive deskilling associated with automated GenAI use, the program design intentionally emphasized critique and judgment over correctness. At the start of the reflection phase, participants were informed that activities were intended to surface pedagogical reasoning, limitations, and ethical considerations rather than to produce optimal outputs. Reflection was facilitated using a structured yet adaptive approach, with participants discussing their generated artifacts in a group setting and articulating considerations related to accuracy, pedagogical alignment, contextual constraints, and responsible implementation. This design foregrounded educator judgment and critical appraisal, positioning GenAI as an assistive resource rather than a substitute for pedagogical decision-making. Faculty completed an online preintervention survey (PreIS) at workshop 1, postintervention survey (PostIS) at workshop 5, and a follow-up survey (FUS) 2 weeks later to capture self-reported learning and intended implementation of GenAI-supported teaching practices.

The workshops were designed as applied and interactive sessions, with learning occurring primarily through guided exploration, structured activities, and facilitated reflection. During each workshop, participants were asked to complete structured activity worksheets to document GenAI prompts, generated artifacts, and reflections on their learning. No required readings or formal homework assignments were assigned between sessions, although participants were encouraged to independently experiment with GenAI tools in their teaching contexts.

### Theoretical Framework

The PDGenAI-P program was grounded in ELT [25,26] and the TPACK [3,24] framework. ELT emphasizes learning through iterative cycles of experience, reflection, conceptualization, and experimentation, supporting active engagement and reflective sense-making in professional learning contexts. ELT progresses from experience to action with 4 phases ordered as experiencing (Concrete Experience), reflecting (Reflective Observation), thinking (Abstract Conceptualization), and acting (Active Experimentation).

**Figure 2.**
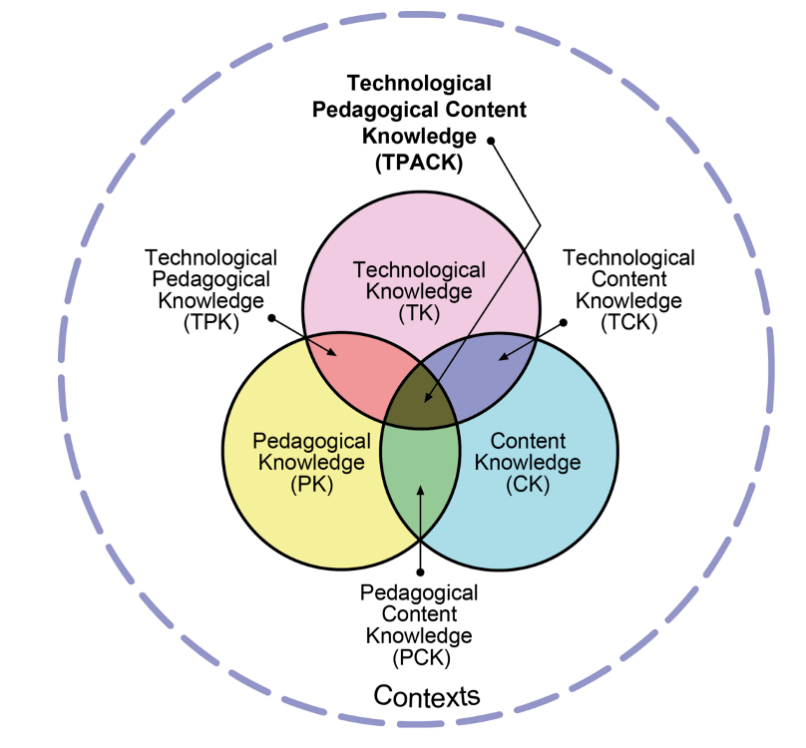
The Technological Pedagogical Content Knowledge framework and its knowledge components (reproduced with permission from the publisher, © 2012 by tpack.org).

TPACK identifies the intersection of technological, pedagogical, and content knowledge essential for effective technology integration in education [24]. The TPACK framework posits that effective technology integration requires a synergistic understanding of 3 primary forms of knowledge, that is, Content Knowledge, Pedagogical Knowledge, and Technological Knowledge. The TPACK framework is built on 7 domains of educator knowledge (Figure 2) that form the basis of good teaching [24]. These are Technological Knowledge, Content Knowledge, Pedagogical Knowledge, Pedagogical Content Knowledge, Technological Content Knowledge, and Technological Pedagogical Knowledge, with the central idea being TPACK.

Although TPACK was originally developed in the context of school-based education [24], subsequent scholarship has extended its application to higher education [27] and health professions education [28], including faculty development and clinical teaching contexts [29-31]. In these settings, TPACK has been used to examine how disciplinary expertise, pedagogical reasoning, and technological fluency intersect in professional education environments where instructional decisions carry high-stakes implications. This broader application supports its relevance for medical faculty navigating integration of emerging technologies such as GenAI.

**Figure 3.**
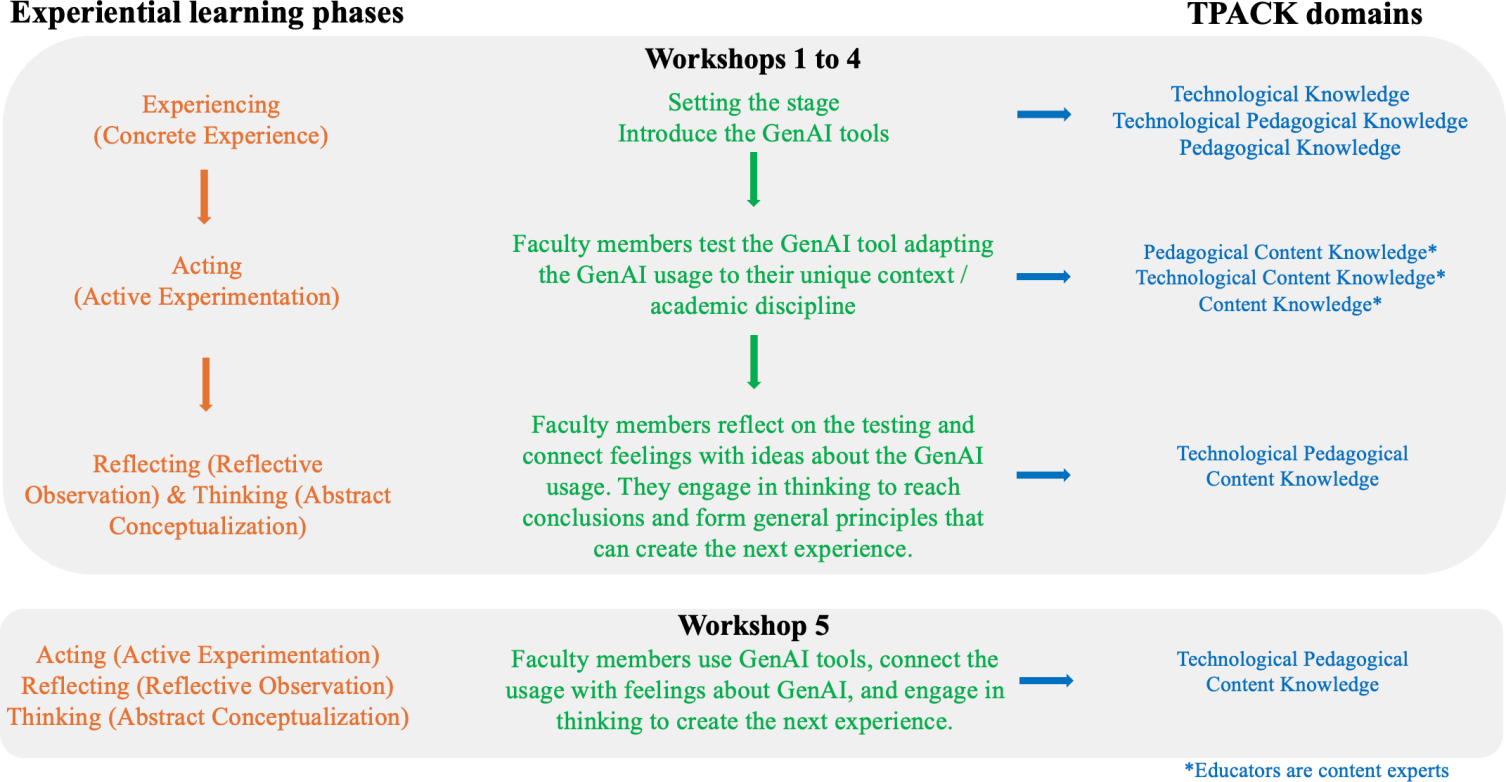
Professional Development in Generative Artificial Intelligence for Pedagogy design describing the mapping of experiential learning phases and Technological Pedagogical Content Knowledge domains across workshops. GenAI: generative artificial intelligence.

Figure 3 illustrates the structure and describes the mapping of ELT phases and TPACK domains in the PDGenAI-P workshops. Each workshop embedded an experiential learning cycle, beginning with facilitator-led examples (concrete experience), followed by hands-on experimentation with GenAI tools, and structured group reflection and conceptualization focused on pedagogical alignment, ethical considerations, and contextual constraints. Although ELT traditionally progresses from experience to action with 4 phases ordered as experiencing (Concrete Experience), reflecting (Reflective Observation), thinking (Abstract Conceptualization), and acting (Active Experimentation), the cycle was intentionally restructured in the PDGenAI-P so that action followed immediately after experience to support rapid testing and critique of GenAI outputs.

This design created a dynamic interplay between experimentation, reflection, and conceptual understanding, enabling faculty to iteratively examine, contextualize, and refine GenAI-supported teaching practices within their own disciplinary settings [5]. Participants used a range of GenAI tools across workshops, including ChatGPT, Claude, Microsoft Copilot, Adobe Firefly, and Bing (at the time of delivery). Tool selection was participant-dependent and varied based on access and activity type; most participants primarily used ChatGPT or Claude, with other tools used for specific activities such as image-based tasks. During each workshop, participants were provided with structured activity worksheets designed to scaffold GenAI prompt development, artifact generation, and reflective analysis. The worksheets guided participants to document prompts used, generated outputs, contextual adaptations, and reflections on pedagogical alignment, limitations, and ethical considerations. These worksheets functioned as learning scaffolds rather than assessment tools and were optional. While participants generated tangible pedagogical artifacts (eg, draft lesson plans, assessment items, role-play scripts, and image-based materials), these artifacts and worksheets were not collected for grading, formally evaluated, or systematically analyzed as outcome data.

## Methods

### Study Design and Intervention Setting

This study examined the design, implementation, and short-term outcomes of a faculty PD workshop series—PDGenAI-P at Weill Cornell Medicine-Qatar, a US medical school in Doha, Qatar. The institution offers a 6-year Doctor of Medicine program comprising 2 premedical years followed by 4 years of ME. At the time of the study, the institution employed 83 full-time faculty members [32]. The PDGenAI-P was delivered synchronously online and included faculty from multiple premedical and ME disciplines. Two iterations of the program were implemented between July and August 2024. A mixed methods design was used, with quantitative and qualitative data collected concurrently to assess faculty experiences, learning processes, and intended near-term application of GenAI tools. This approach enabled the integration of descriptive quantitative findings with qualitative analysis of workshop transcriptions, facilitator memos, and facilitator theoretical notes to contextualize how faculty engaged with GenAI within their pedagogical settings.

### Participants

Participants were recruited from the faculty body at Weill Cornell Medicine-Qatar.. The institution delivers a US medical curriculum and employs a diverse, multinational faculty engaged in premedical and medical education and research. Recruitment took place within this institutional setting, targeting faculty members involved in teaching across preclinical and clinical phases of the curriculum. Ten faculty members from Weill Cornell Medicine-Qatar participated in PDGenAI-P. As an exploratory pilot faculty development intervention, the study focused on feasibility and short-term self-reported learning and intended application rather than statistical generalizability.

Recruitment was voluntary and open to all teaching faculty at the institution. Faculty across multiple disciplines were invited via targeted email outreach to ensure representation from a range of premedical and ME subspecialities. The invitation email or recruitment letter outlined the purpose of the study, participation requirements, and the voluntary nature of involvement. Participation was not incentivized. Participants therefore represent a self-selected sample of faculty interested in GenAI and PD, rather than a statistically representative sample of all faculty at the institution. Written informed consent was obtained prior to participation in the workshops.

### Data Collection

Quantitative and qualitative data were collected concurrently during the PDGenAI-P [33,34]. Quantitative data were collected from an online PreIS and online PostIS with open-ended questions administered at the start and end of the PDGenAI-P, and an online FUS which was sent via email, 2 weeks after the PDGenAI-P was completed. Surveys were administered electronically using Qualtrics survey software (Qualtrics).

The PreIS primarily focused on faculty TPACK for GenAI as part of a dissertation study; only demographic information and baseline self-reported data on GenAI familiarity were used to contextualize participant experience levels for this manuscript. Detailed TPACK analyses are reported elsewhere and are outside the scope of this manuscript. Participants’ familiarity with GenAI tools was identified through workshop self-descriptions and facilitator observations. It was then confirmed using a single self-rated familiarity item in the PreIS, in which participants selected 1 of 5 levels ranging from “no knowledge” to “expert level,” based on their understanding and use of GenAI tools. Participants were also invited to provide open-text examples of GenAI use.

PostIS and FUS included both quantitative items and open-ended questions inviting reflections on learning experiences, challenges, and recommendations. Likert-scale items in the PostIS assessed perceptions of program quality (content, organization, relevance, and overall quality), and in the FUS, assessed self-reported confidence in applying GenAI concepts to teaching practice and perceived shifts in perspective. Open-ended questions captured reflections on learning experiences, challenges, and intended application of GenAI in teaching. Confidence and perspective change were each measured using single-item 5-point ordinal scales. The study did not include objective competency-based assessments or validated multi-item scales for these constructs. A 2-week follow-up interval was selected to assess early retention, continued experimentation, and intended near-term application of GenAI following program completion. This interval was chosen to balance feasibility with the opportunity for participants to independently engage with GenAI in their teaching context shortly after the intervention. Follow-up results are therefore interpreted as short-term indicators rather than evidence of sustained integration or long-term behavior change. The survey instruments are provided in Multimedia Appendix 2.

Qualitative data sources included transcriptions of workshop discussions. Analytic sensibilities were informed by ongoing reflexive memoing and maintenance of theoretical notes throughout the design, facilitation, and implementation of the PDGenAI-P workshops. These notes were used to document emerging pedagogical considerations, participant responses, and facilitator reflections.

### Quantitative Data Analysis

Descriptive statistics were used to summarize quantitative data. Given the exploratory pilot design and small sample size, quantitative findings are reported descriptively and interpreted as indicators of participant perceptions rather than inferential outcomes. In the absence of a comparison group, the study was not intended to isolate the effects of specific program components or establish causal attribution. Findings reflect participant-reported perceptions and analytically derived patterns across data sources rather than demonstrable causal impact. While PreIS and PostIS were administered, these instruments primarily captured self-reported perceptions rather than objective pretest or posttest measurement of pedagogical skill. Quintile rankings reflect ordinal self-assessment and should not be interpreted as validated measures of learning gain.

### Qualitative Data Analysis

Qualitative analysis followed a combination of deductive and inductive coding [33,35] drawing on constructs from TPACK and ELT while allowing themes to emerge. Initial deductive coding using the TPACK framework was conducted to sensitize analysis to technological, pedagogical, and disciplinary considerations relevant; however, detailed TPACK analyses are outside the scope of this manuscript. Inductive coding was subsequently used to capture patterns emerging from participant reflections and workshop interactions. While thematic analysis was initially conducted, a narrative analytic approach was ultimately used to more authentically represent the experiential and temporal progression of the workshops and participants’ articulated learning and intentions [36]. All qualitative analysis was conducted by the corresponding author using Dedoose (SocioCultural Research Consultants, LLC), a cloud application for managing, analyzing, and presenting qualitative and mixed methods research data [5].

Rigor and trustworthiness were supported through reflexive memoing and theoretical note-taking across design, implementation, and analysis; iterative cycling between data, interpretations, and analytic approaches; triangulation across coding, thematic, and narrative analyses; and member checking of interpretive summaries with the principal investigator and site principal investigator to enhance credibility.

### Data Integration

Findings were derived through an integrated analysis in which quantitative and qualitative data were collected concurrently and analyzed separately. Quantitative data were analyzed descriptively, and qualitative data were analyzed using deductive and inductive approaches, alongside narrative analysis. Integration occurred at the interpretation stage, where quantitative results contextualized participant-reported perceptions, while qualitative data provided depth, context, and explanatory insights into how participants engaged with GenAI across workshops. Both data strands were interpreted together to identify areas of convergence, complementarity, and divergence. These were synthesized to generate insights on faculty experiences, learning processes, and intended application. Facilitator memos and theoretical notes were reviewed to triangulate findings, enrich interpretation, and capture iterative changes in faculty engagement.

### Ethical Considerations

The study received approval from the institutional review boards (IRBs) of both Arizona State University (IRB# STUDY00020020) and Weill Cornell Medicine-Qatar (IRB# 24‐00027) and was conducted as part of the corresponding author’s doctoral research. All participants received written information about the study’s purpose, procedures, voluntary nature, and data confidentiality. Informed consent was obtained prior to participation. Surveys were anonymous. All participant responses were confidential. Given the study’s focus on GenAI tools, additional measures were taken to address ethical considerations specific to GenAI use. Faculty were informed about the limitations of GenAI tools, including potential hallucinations, bias in training data, and risks associated with overreliance on AI-generated content. During workshops, participants were encouraged to critically evaluate GenAI outputs and apply disciplinary expertise to verify accuracy. No student data or patient-related information was used at any point. The study adhered to ethical guidelines for educational research and maintained compliance with institutional data governance policies throughout the project.

## Results

### Participants Characteristics

Ten faculty members participated in the PDGenAI-P workshop series. Participants represented a range of academic disciplines, including anatomy, biochemistry, biology, biophysics, biostatistics, chemistry, medical humanities, pathology, public health, research methodology, and physics. In terms of professional experience, 5 (50%) participants identified as senior faculty with more than 10 years of professional experience, 2 (20%) were midcareer (5‐10 years), and 3 (30%) were junior faculty with fewer than 5 years of experience. Six (60%) participants had been employed at the institution for more than 10 years. Participants reported varying levels of prior familiarity with GenAI tools. This distribution reflects participation from faculty at varying career stages and levels of teaching experience. This diversity enhanced disciplinary breadth but limited claims of representativeness or homogeneity.

### Workshop-Level Progression

This subsection presents findings derived from narrative analysis of workshop transcripts, facilitator memos, and theoretical notes from the PDGenAI-P. Across the 5 PDGenAI-P workshops, narrative analysis indicated a developmental progression in faculty engagement with GenAI tools, moving from exploratory use toward more intentional pedagogical application and early integration within teaching contexts. In early sessions, faculty experimented with text-based GenAI tools with curiosity but expressed uncertainty regarding reliability and accuracy. Across early workshop discussions and reflections, faculty described GenAI outputs as often generic or incomplete without disciplinary oversight, prompting recalibration of expectations about GenAI’s capabilities and limitations.

As workshops progressed, narrative comparison across sessions showed that faculty applied GenAI to specific pedagogical tasks, including lesson planning and case scenario design (ChatGPT/Claude/Copilot), assessment design (ChatGPT/Claude), image generation (ChatGPT/Adobe Firefly/Bing), and simulation-based role-play (ChatGPT/Claude/Copilot). Narrative analysis of participant reflections indicated increasing confidence with prompt refinement and a more nuanced understanding of tool limitations, especially related to hallucinations, contextual misalignment, and training data bias. Across successive workshop transcripts, faculty discourse shifted from tool operation to deeper pedagogical reasoning as they considered when, why, and how GenAI could meaningfully enhance learning. Cross-session analysis showed faculty applying GenAI tools (primarily ChatGPT and Claude) to address discipline-specific instructional challenges. Workshop transcripts showed greater fluency in faculty adapting GenAI outputs, openly discussed ethical considerations, and describing GenAI as a potential “copilot” to augment rather than replace, educator expertise. Narrative analysis of workshop discussions showed ethical discussions centered on tool hallucinations, data bias, plagiarism, and the importance of pedagogical intent. They also reinforced the need for authenticity, bias mitigation, and human oversight. This phase reflected consolidation of learning, identified through synthesis of participant reflections and application-focused discussions, as faculty connected GenAI capabilities with their pedagogical goals. Across all workshops, faculty reflections illustrated an evolving mindset from exploratory use to critical, discipline-specific integration. Facilitator memos corroborated analytically derived patterns, documenting similar shifts in faculty discourse from tool operation to pedagogical alignment and ethical reflection. Findings in this subsection reflect qualitative interpretation of participant discussions and reflections rather than objective measures of behavior change.

### Workshop Experiences and Perspectives (PostIS)

Quantitative PostIS results are reported descriptively to summarize participant perceptions of program quality and relevance. All participants (n=10) completed the PostIS. Faculty rated workshop content, organization, relevance, and overall quality of the program as either above average (n=4, 40%) or excellent (n=6, 60%), with no responses below these categories (Table 1). Relevance was rated as above average by 5 (50%) participants or excellent by 5 (50%) participants. Qualitative feedback further supported these ratings. The faculty described workshops as “directly responding to our needs” and praised the logical sequencing and hands-on structure. Faculty reported that immediate experimentation facilitated meaningful learning, and several highlighted newly discovered applications for GenAI in their teaching and research. One faculty member commented, “This workshop opened my eyes to all the resources in GenAI. I will definitely use GenAI to enhance my teaching and research.” Constructive suggestions included allowing more time for reflection and offering temporary access to premium GenAI tools to mitigate limitations of freeware versions.

**Table 1.** Participant ratings of the PDGenAI-P^a^ workshops (PostIS^b^; N=10)^c^.

Workshop domain	Very poor, n (%)	Below average, n (%)	Average, n (%)	Above average, n (%)	Excellent, n (%)
Workshop content	0 (0)	0 (0)	0 (0)	4 (40)	6 (60)
Workshop organization	0 (0)	0 (0)	0 (0)	4 (40)	6 (60)
Workshop relevance	0 (0)	0 (0)	0 (0)	5 (50)	5 (50)
Overall workshop quality	0 (0)	0 (0)	0 (0)	4 (40)	6 (60)

a PDGenAI-P: Professional Development in Generative Artificial Intelligence for Pedagogy.

b PostIS: postintervention survey.

c Ratings were based on a 5-point Likert scale (1=Very poor to 5=Excellent).

### Knowledge and Confidence (FUS)

Given the exploratory nature of the FUS and small sample size, results are reported as descriptive indicators of early confidence rather than inferential outcomes. Five (50%) faculty members completed the FUS 2 weeks after PDGenAI-P. This construct assessed self-reported readiness to apply GenAI-related skills in teaching practice. As shown in Table 2, faculty reported varying levels of confidence in applying workshop concepts to their teaching: 2 (40%) were “very confident,” 2 (40%) were “moderately confident,” and 1 (20%) reported being “slightly confident.” No faculty selected “not confident.” In open-ended responses, faculty attributed increased confidence to hands-on experimentation, iterative prompt refinement, and opportunities for guided reflection. One faculty member commented, “The workshop introduced us to different and very interesting GenAI tools. I am experimenting with these tools and learning to apply them in my daily teaching,” highlighting the importance of continued practice.

**Table 2.** Self-reported confidence in applying PDGenAI-P^a^ concepts or skills in pedagogical practice (FUS^b^; N=5)^c^.

Confidence level	Values, n (%)
Not at all confident (I do not feel prepared to apply the concepts or skills in my teaching practice).	0 (0)
Slightly confident (I feel somewhat prepared but would need more guidance or practice before applying these concepts or skills in my pedagogical practice).	1 (20)
Moderately confident (I feel somewhat prepared and might try to apply some of the concepts or skills in my pedagogical practice).	2 (40)
Confident (I feel prepared and confident in my ability to apply most concepts or skills learned in my pedagogical practice).	0 (0)
Very confident (I feel fully prepared and very confident in my ability to seamlessly integrate all concepts or skills learned in my pedagogical practice).	2 (40)

a PDGenAI-P: Professional Development in Generative Artificial Intelligence for Pedagogy.

b FUS: follow-up survey.

c Confidence was assessed on a 5-point Likert scale ranging from 1=Not at all confident to 5=Very confident.

### Shift in Perspectives on GenAI in Teaching and Learning (FUS)

This FUS construct asked participants to self-rate perceived changes in their understanding of GenAI’s role in teaching and learning. Among respondents (n=5), 3 (60%) participants reported a moderate perceived change, 1 (20%) participant reported a slight change, and 1 (20%) participant reported a substantial perceived change (Table 3). Given the small number of responses and the self-reported nature of the item, these results reflect subjective perceptions rather than objectively measured change. Qualitative analysis of open-ended follow-up responses provided additional context for these participants’ self-reported perceived changes shifts. Faculty who indicated moderate perceived change described gaining new insights into the pedagogical applications of GenAI beyond text generation. One respondent who indicated a substantial perceived change described beginning to use GenAI in planning small-group activities and problem-based learning sessions. The faculty who reported complete transformation noted adopting GenAI to redesign small-group activities, laboratories, and problem-based learning sessions. The faculty member observed, “After this workshop, I’m using GenAI to plan PBL sessions and syllabus design. It has changed how I think about lesson preparation.” These comments reflect participant-reported experiences rather than independently verified changes in instructional practice.

**Table 3.** Self-reported perceived change in faculty perspectives on teaching and learning with GenAI^a^ tools (FUS^b^; N=5)^c^.

Change level	Values, n (%)
No change at all (My perspectives have not changed after attending the workshops. I maintain the same views as I did before).	0 (0)
Slight change (There has been a minor shift in my perspectives. I see a few things differently but largely hold the same views).	1 (20)
Moderate change (I have gained new insights that have changed my views to some extent).	3 (60)
Considerable change (I have adopted many new views and approaches).	0 (0)
Complete transformation (The workshops have changed my approach and understanding of GenAI^d^ in teaching and learning).	1 (20)

a GenAI: generative artificial intelligence.

b FUS: follow-up survey.

c Perceived change was assessed on a 5-point scale ranging from 1=No change to 5=Complete transformation.

d GenAI: generative artificial intelligence.

### Intended Implementation Strategies and Good Practices (FUS)

This FUS construct item asked faculty to indicate how they intended to use GenAI in teaching and administrative workflows. Among respondents (n=5), all 5 (100%) participants reported intentions to use GenAI to support lesson planning, instructional design, and assessment development, including multiple-choice questions and United States Medical Licensing Examination–style questions (Table 4). Four participants (80%) indicated plans to use GenAI to develop case scenarios for project-based learning and to visualize complex learning material. Fewer respondents reported intentions to define responsible use practices (n=2, 40%), initiate ethical discussions (n=1, 20%), or adapt materials for personalized learning (n=1, 20%). These results reflect short-term self-reported intentions and should be interpreted as exploratory indicators of anticipated application rather than evidence of implementation.

**Table 4.** Faculty-intended utilization of GenAI^a^ in teaching and learning activities (FUS^b^; N=5).

Implementation strategies	Values, n (%)
Create case scenarios to facilitate project-based learning.	4 (80)
Create a lesson plan, interactive activities and a rubric using a backward design model.	5 (100)
Visualize learning material.	4 (80)
Create various types of assessments (MCQs^c^ USMLE^d^ style, formative etc) based on the lesson plan and rubric.	5 (100)
Adapt learning material to create personalized learning experiences.	1 (20)
Critically analyze content GenAI.	1 (20)
Define responsible use of GenAI in your settings.	2 (40)
Initiate discussions of responsible and ethical use of GenAI in your classroom.	1 (20)
Manage administrative tasks.	1 (20)

a GenAI: generative artificial intelligence.

b FUS: follow-up survey.

c MCQs: multiple-choice questions.

d USMLE: United States Medical Licensing Examination.

### Anticipated Concerns and Support Needs (FUS)

The FUS also asked participants to describe anticipated challenges and additional support required for GenAI implementation (Table 5). Among respondents (n=5), 3 (60%) described minor anticipated obstacles including limited institutional guidance, access constraints, or the need for continued practice. One (20%) reported no anticipated obstacles, and 1 (20%) anticipated more substantial challenges requiring additional training or structural support. These results reflect perceived expectations rather than verified implementation barriers. They provide preliminary insight into areas where ongoing faculty development or institutional support structures may warrant consideration.

**Table 5.** Faculty levels of concern regarding application of GenAI^a^ tools (FUS^b^; N=5)^c^.

Level of concern	Values, n (%)
Very concerned (I am highly concerned about several major obstacles and feel I need additional support to apply my learning).	0 (0)
Fairly concerned (I foresee several obstacles and will need to seek solutions to overcome them).	1 (20)
Moderately concerned (I am worried about potential obstacles and am considering strategies to address them).	0 (0)
Mildly concerned (I have slight concerns that there might be minor obstacles, but I feel well equipped to manage them).	3 (60)
Not concerned at all (I am completely confident in my ability to apply the learning and resolve any obstacles that I might encounter).	1 (20)

a GenAI: generative artificial intelligence.

b FUS: follow-up survey.

c Level of concern was assessed on a 5-point scale ranging from 1 = Very concerned to 5 = Not concerned at all.

Faculty also identified areas of support that they perceived would enhance their ability to apply PDGenAI-P strategies in practice (Table 6). Among respondents (n=5), 4 (80%) indicated interest in consulting with GenAI experts and participating in peer discussion forums, and 3 (60%) expressed interest in additional workshops to deepen understanding. Participants also requested curated guides (n=3, 60%), institutional navigation support (n=3, 60%), and step-by-step instructions for integrating GenAI into teaching (n=2, 40%). These results should be interpreted as exploratory indicators of anticipated support requirements rather than definitive implementation gaps.

**Table 6.** Faculty-perceived support needs (FUS^a^; N=5).

Description of support	Values, n (%)
Step-by-step instructions for integrating PDGenAI-P^b^ strategies into teaching practices.	2 (40)
Opportunities to discuss specific challenges and questions with GenAI^c^ experts.	4 (80)
Forums for exchanging ideas and experiences with other educators applying GenAI.	4 (80)
Additional workshops or training sessions to deepen understanding and address challenges.	3 (60)
A customized collection of online guides and resources for further exploration.	3 (60)
Guidance on navigating institutional policies and obtaining support.	3 (60)

a FUS: follow-up survey.

b PDGenAI-P: Professional Development in Generative Artificial Intelligence for Pedagogy.

c GenAI: generative artificial intelligence.

### Summary of Findings

The integrated quantitative and qualitative findings reflect high participant-reported satisfaction, perceived increases in confidence, and articulated intentions to integrate GenAI into teaching contexts. These results derive from a small pilot cohort and rely on self-reported perceptions rather than objective measures of instructional change. As such, they should be interpreted as exploratory indicators of early engagement. The findings suggest that a theory-informed, experiential workshop series may serve as a feasible entry point for supporting faculty reflection and initial GenAI experimentation in ME, while underscoring the need for longitudinal evaluation and institutional reinforcement.

## Discussion

### Principal Findings

This pilot mixed methods study explored the design and short-term participant-reported outcomes of PDGenAI-P, a faculty development workshop series focused on integrating GenAI into ME teaching. Findings suggest that a theory-informed, experiential workshop format may support medical educators in developing the initial knowledge, perceived confidence, and reflection needed to integrate GenAI tools responsibly into their teaching. Qualitative analysis suggested that structured, hands-on engagement combined with facilitated reflection may support medical educators in moving from exploratory tool use toward more intentional pedagogical application. Participants reported increased self-reported confidence and articulated intended strategies for using GenAI in lesson planning, assessment design, visualization, and case-based instruction. These findings reflect perceived learning and engagement rather than independently verified changes in teaching practice. They offer preliminary insight into how short, structured faculty development may initiate reflective engagement with GenAI in ME contexts.

In relation to scholarship on emerging educational technologies in education and health professions training, several patterns warrant consideration. The observed engagement aligns with literature suggesting that faculty benefit from experiential, context-sensitive approaches when adopting rapidly evolving educational technologies [37-39]. Structured opportunities for experimentation, guided critique, and peer dialogue appear important in supporting thoughtful integration of digital tools [40,41]. At the same time, the imbalance observed between strong intentions for content creation and comparatively lower intention for explicit ethical operationalization mirrors concerns in the broader GenAI in-education literature that early adoption may prioritize efficiency gains over critical or reflexive practice [42,43]. These patterns underscore the importance of longitudinal scaffolding beyond initial exposure.

Participants’ reported satisfaction and articulated applications suggest that short, theory-informed workshop series may create space for educators to reflect on how GenAI intersects with disciplinary expertise and instructional decision-making. Rather than positioning GenAI as a substitute for educator judgment, the workshop design emphasized structured experimentation and facilitated reflection in supporting intentional, context-sensitive integration. Participants frequently attributed their learning to the experiential, hands-on structure of the workshop series, particularly the opportunity to test GenAI outputs within their own disciplinary contexts. Their reflections suggest that iterative experimentation combined with facilitated dialogue may help educators critically appraise both the affordances and limitations of GenAI tools.

The workshop series was informed by ELT and the TPACK framework, and the observed patterns of engagement were consistent with the principles underlying these models [3,24-26]. Structuring activities around cycles of experimentation and reflection, while explicitly connecting technological tools to pedagogical and disciplinary considerations, appeared to create opportunities for faculty to examine how GenAI might intersect with their existing expertise. Rather than emphasizing tool proficiency alone, the approach situated GenAI within broader pedagogical reasoning, reinforcing the centrality of professional judgment in ME.

Facilitator reflections across the 2 PDGenAI-P iterations suggest that adaptive facilitation played an important role in shaping participant engagement. Modifications such as shortening introductory content, increasing time for hands-on exploration, and shifting from structured guidance to flexible experimentation were implemented directly in response to faculty feedback. These adjustments were associated with more active participation and extended reflective dialogue during subsequent sessions. Given that participants’ diverse disciplinary priorities and limited time, responsiveness to context appeared important. However, the extent to which facilitation strategies alone can influence sustained pedagogical change remains uncertain, reinforcing the need for continued institutional and longitudinal support.

The PostIS and FUS results reflect short-term, self-reported perceptions rather than objective evidence of sustained pedagogical change. Faculty described increased confidence in applying GenAI tools and perceived shifts in how they conceptualized and planned instructional activities. However, these responses were collected 2 weeks postintervention and were based on a limited subset of participants (n=5) warranting cautious interpretation. Reported intended uses of GenAI focused on assessment design, lesson planning, visualization of learning materials, and the creation of case scenarios, suggesting primarily applied and discipline-specific integration. Ethical considerations, such as bias and hallucinations, were acknowledged in reflections; suggesting awareness of responsible GenAI use, although intentions to operationalize these practices were less consistently articulated. This highlighted a potential gap between awareness and explicit implementation planning. These patterns should be interpreted as early indicators of engagement rather than a confirmation of durable transformation in teaching practice. Intention to apply GenAI and confidence in doing so do not necessarily translate into consistent implementation, nor do they guarantee long-term integration. Longitudinal evaluation and behavioral measures would be necessary to determine whether reported shifts are sustained over time and embedded into routine pedagogical practice.

An imbalance was observed in the FUS responses, with faculty reporting strong intentions to use GenAI for content creation and assessment design but lower intention to engage in critical evaluation of GenAI outputs, initiate ethical discussions, or define responsible use. Although based on a small sample (n=5), this pattern aligns with broader educational technology literature suggesting that early adoption is often characterized by instrumental, efficiency-oriented uses before more critical, reflective, and ethically grounded practices are operationalized [23,43,44]. In ME, where ethical reasoning and professional judgment are central, this finding underscores the importance of continued scaffolding and longitudinal faculty development to support translation of ethical awareness into explicit pedagogical practice, rather than relying on initial exposure alone [6,45,46]. Awareness of risks such as bias and hallucinations does not necessarily translate into structured pedagogical practices that foreground responsible use [7,43], suggesting that faculty development initiatives should move beyond exposure and skill-building toward explicit modeling and reinforcement of critical appraisal and responsible use.

The integration of GenAI in ME raises important risks, including overreliance on artificial intelligence–generated outputs, automation bias, and potential cognitive deskilling, if learners or educators defer uncritically to generated content. Automation bias, well described in clinical decision support contexts, can lead individuals to privilege automated recommendations even when flawed, contributing to errors of commission or omission and reducing vigilance in information seeking [47]. Parallel concerns have emerged in ME scholarship where uncritical or habitual reliance on emerging technology such as GenAI may promote cognitive offloading and undermine cognitive autonomy, particularly when use is not accompanied by explicit pedagogical scaffolding and reflective appraisal [11,48]. Within the workshop series, risks such as hallucinations, bias, and overreliance were explicitly discussed and participants were encouraged to verify outputs against disciplinary standards and position GenAI as a “copilot” rather than a substitute for professional judgment. Nevertheless, short-term faculty development cannot eliminate structural risks associated with automation bias or cognitive offloading. In ME, where diagnostic reasoning, ethical judgment, and patient safety are foundational [49,50], sustained institutional alignment and longitudinal reinforcement are necessary to ensure that GenAI augments rather than diminishes professional competence [7,43].

In summary, these findings suggest that faculty development for GenAI integration should move beyond technical proficiency and efficiency oriented use toward structured opportunities for experimentation, critical and ethical evaluation, and discipline-specific pedagogical alignment. Early indicators of satisfaction and self-reported confidence should not be interpreted as comprehensive integration but rather as initial stages in a longer developmental process. Particularly in ME, where ethical judgment and professional accountability are central, short-term faculty development may initiate experimentation and reflection but cannot complete the work of embedding GenAI into teaching in critically reflective and ethically explicit ways.

### Implications for Faculty Development

Several implications emerge for the design and scaling of GenAI-focused faculty development in ME. Iterative hands-on experimentation supported by structured reflection appears important for enabling faculty to evaluate GenAI tools within their disciplinary contexts. Adaptive facilitation grounded in learning theory may enhance relevance and responsiveness to participant needs [26]. Longitudinal structures progress from foundational prompting strategies to more advanced applications, such as simulation design, or personalized learning development may further support pedagogical fluency [16,17]. Institutional infrastructure, including curated repositories of vetted prompts, exemplar lesson plans, and structured use cases, may reduce implementation barriers and promote consistent practice [16,17,43]. Equally important are communities of practice and peer-mentoring structures that foster sustained dialogue, shared problem-solving, and ethical reflection as GenAI tools evolve [51]. Attention to responsible use, including bias detection, output verification, and transparency, remains essential to align GenAI integration with professional standards in ME.

### Limitations

This study has several limitations. First, reported outcomes are based on self-reported confidence, perceived shifts in thinking, and intended implementation strategies rather than direct observation of enacted teaching practice. Self-reported measures are also susceptible to social desirability bias and overestimation of competence, particularly immediately following a PD intervention. The study did not include classroom observations, external evaluation of instructional artifacts, structured rubrics, performance-based assessments, behavioral indicators of pedagogical change, or longitudinal follow-up beyond 2 weeks. Ethical and critical dimensions of GenAI use were explicitly discussed throughout the workshops but were not assessed as measurable competencies. Consequently, expressed awareness of ethical concerns should not be interpreted as demonstrated competence in ethical enactment.

Second, the study design limits causal inference. The intervention was not evaluated against alternative faculty development approaches, and no comparison or control group was included. Because component-level analyses were not conducted, it is not possible to determine which elements of the workshop series may have contributed to reported changes, nor to attribute observed patterns solely to the intervention itself. Although a PreIS was administered, the study did not use objective pretest or posttest assessments of specific GenAI pedagogical competencies, nor did it include a comparison group. Outcomes therefore reflect descriptive, self-reported perceptions rather than verified changes in teaching practice. Instructional artifacts generated during workshops were not formally evaluated, and teaching practices were not observed. The quantitative tables represent descriptive summaries of participant perceptions and intended use. The sample included faculty from multiple disciplines and career stages, which enhanced diversity of perspectives but limited homogeneity and statistical generalizability.

Third, the follow-up period was limited to 2 weeks, and only half of the participants (n=5) completed the FUS. This reduces the breadth of longitudinal perspectives available and restricts insight into sustained integration and introduces potential response bias. Confidence and intention to use GenAI do not necessarily translate into consistent implementation, or guarantee long-term pedagogical transformation. Finally, the small, self-selected sample (n=10) and single-institution context limit transferability to other ME settings. Although baseline GenAI familiarity was captured, confidence in GenAI use was not independently measured prior to the intervention. Quantitative findings are based on descriptive measures only, and no inferential statistical analyses were conducted given the pilot design.

### Future Work

This pilot study was designed to examine feasibility and early indicators of engagement rather than to establish effectiveness or long-term impact. Future research should incorporate longitudinal designs; objective, performance-based measures; multisite implementation; and structured evaluation of ethical competence to determine whether reported shifts translate into sustained and observable transformation in ME practice. Despite these limitations, the iterative design and adaptive facilitation of PDGenAI-P provided preliminary insight into how an experiential, theory-informed GenAI faculty development workshop series may support early engagement and critical reflection. Future research with larger samples, multisite collaborations, and longitudinal evaluation will be important to assess broader applicability and sustained impact on instructional practice. Future work should examine how PDGenAI-P might evolve beyond a pilot workshop series into a scalable and institutionally integrated faculty development initiative. Offering the program as an accredited Continuing Medical Education or Continuing Professional Development activity may acknowledge the depth of pedagogical and ethical engagement required for responsible GenAI use in ME. A longitudinal structure that progresses from foundational prompting strategies to advanced applications such as GenAI-supported simulation design may further develop faculty fluency and pedagogical judgment. Developing a structured implementation guide could facilitate cross-institutional adoption while supporting contextual adaptation rather than replication.

Sustainable integration will likely depend on institutional infrastructure. Centralized repositories of vetted prompts, exemplar lesson plans, and instructional resources, alongside integration within existing learning management systems, may reduce logistical barriers. Equally important are social learning structures, such as interdisciplinary communities of practice and peer-mentoring structures, which may support ongoing critical dialogue, shared problem-solving, and ethical reflection, reducing the sense of isolation that often accompanies rapid technological change. Further empirical evaluation will be necessary to determine how such structures influence sustained and responsible GenAI integration in ME contexts.

### Conclusions

This exploratory pilot study provides preliminary insight into the design and short-term reception of a theory-informed faculty development workshop series PDGenAI-P, focused on GenAI integration in ME. Findings reflect participant-reported engagement, perceived confidence, and early intentions rather than verified changes in teaching practice. While the workshop series appeared to initiate experimentation and reflection, sustained pedagogical and ethical integration will likely require institutional alignment, longitudinal reinforcement, and explicit modeling of responsible GenAI facilitation. Future research should incorporate objective measures of instructional change, including evaluation of implemented teaching artifacts, and long-term follow-up to determine whether early shifts translate into durable transformation in ME practice.

## Supplementary material

10.2196/89815 Multimedia Appendix 1 Overview of Professional Development in Generative Artificial Intelligence for Pedagogy workshops and scaffolded generative artificial intelligence prompts.

10.2196/89815 Multimedia Appendix 2 Preintervention, postintervention, and follow-up survey instruments.

## Abbreviations

ELT: Experiential Learning Theory
FUS: follow-up survey
GenAI: generative artificial intelligence
IRB: institutional review board
ME: medical education
PD: professional development
PDGenAI-P: Professional Development in Generative Artificial Intelligence for Pedagogy
PostIS: postintervention survey
PreIS: preintervention survey
TPACK: Technological Pedagogical Content Knowledge
