# Supplementary material for: Design and Evaluation of a Faculty Development Workshop Series on Integrating Generative Artificial Intelligence in Medical Education: Mixed Methods Pilot Study
Source: JMIR Med Educ. 2026 May 6;12:e89815. doi: 10.2196/89815 (PMC13148327; doi:10.2196/89815)
Supplement: Multimedia Appendix 1 [file mededu-v12-e89815-s001.docx]

**Multimedia Appendix 1**

PDGenAI-P learning objectives:

(1) Analyze key characteristics, capabilities, and limitations of GenAI within medical education contexts.

(2) Evaluate transparent and responsible GenAI practices that foreground human expertise and educator decision making.

(3) Design pedagogically sound learning activities by integrating GenAI with medical educators’ disciplinary and instructional expertise.

(4) Evaluate ethical and pedagogical challenges associated with GenAI use in medical education teaching and exercise professional judgment in mitigating them.

(5) Value human expertise, confidence, and professional judgment when engaging with GenAI as an assistive, rather than substitutive, pedagogical resource.

| Workshop | Example Activity /  Pedagogical Focus | GenAI Tools Used | Example Scaffolded Prompt | Experiential Learning Phase | Intended Learning Outcomes |
| --- | --- | --- | --- | --- | --- |
| Workshop 1  (Case scenario / lesson plans) | Introduction to GenAI? | - | - | Concrete Experience | 1 |
| Workshop 1  (Case scenario / lesson plans) | Create a case scenario | ChatGPT 3.5/4o | You are a medical educator teaching premedical students with an expertise in community medicine.  Generate a unique outbreak scenario with patient zero, transmission rates, symptoms, and demographic data in no more 150 words.  Formulate a hypothesis for this scenario.  Test this hypothesis.  Critique the hypothesis testing. | Concrete Experience | 1, 3 |
| Workshop 1  Case scenario / lesson plans) | Create a case scenario | ChatGPT 3.5/4o | You are a medical educator teaching <<**enter medical program**>> students with an expertise in <<**enter specialty**>>. Generate a unique outbreak scenario with patient zero, transmission rates, symptoms, and demographic data in <<**enter number of words**>>.  Optional: Add a brief introduction to the prompt that would help set the stage for students. Explain why understanding outbreak scenarios is important in this specialty.  Formulate a hypothesis for this scenario in <<**enter number of words**>>  Test this hypothesis in <<**enter number of words**>>  Critique the hypothesis testing in <<**enter number of words**>> | Active Experimentation | 1, 3, 5 |
| Workshop 1  (Case scenario / lesson plans) | Create a case scenario | ChatGPT 3.5/4o | Can I apply this to my teaching? If so, how? | Reflective Observation and  Abstract conceptualization | 2, 4, 5 |
| Workshop 1  (Case scenario / lesson plans) | Create a case scenario | ChatGPT 3.5/4o | Potential challenges?  Ethical implications?   - What stood out to you during the experience? - How did this experience differ from your expectations? - What role did your assumptions and beliefs play as you approached this experience? - What potential challenges do you foresee? - What are the ethical implications? | Reflective Observation and  Abstract conceptualization | 2, 4, 5 |
| Workshop 1  (Case scenario / lesson plans) | Create a lesson plan | Claude 3.5 | You are a professor in a medical school with expert knowledge in biochemistry and pedagogy.  Create a lesson plan using the backward design framework on ‘The Krebs Cycle’ for premedical students, 50 minutes in duration and include learning objectives and 2 interactive activities.  The students have limited knowledge about the topic.  Use Blooms Taxonomy to design the lesson to include a variety of lower order to higher order thinking activities. Indicate which activities use which skills.  Create a rubric for assessing student learning based on the learning objectives.  Create the output in a table format showing learning objectives, assessments and activities.  Evaluate this lesson plan. How could you improve this? Were there any limitations? | Concrete Experience | 1, 3 |
| Workshop 2  (Assessments) | Generate a USMLE style assessment | ChatGPT / Claude | Generate four USMLE style questions on heart failure. | Concrete experience | 1, 3 |
| Workshop 2  (Assessments) | Generate a USMLE style assessment | Claude & ChatGPT | Generate **enter name of style** multiple-choice questions focused on **enter topic**.  Ensure each question includes a **enter context** and appropriate answer choices.    The answer choices should include **enter number** correct answer and **enter number** distractors.  Additionally, provide the correct answer for each question **and an explanation for selecting the wrong answer.** | Active Experimentation | 1, 3, 5 |
| Workshop 2  (Assessments) | Generate a USMLE style assessment | Claude & ChatGPT | Can I apply this to my teaching? If so, how? | Reflective Observation and  Abstract conceptualization | 2, 4, 5 |
| Workshop 2  (Assessments) | Generate a USMLE style assessment | Claude & ChatGPT | Potential challenges?  Ethical implications?   - What stood out to you during the experience? - How did this experience differ from your expectations? - What role did your assumptions and beliefs play as you approached this experience? - What potential challenges do you foresee? - What are the ethical implications? | Reflective Observation and  Abstract conceptualization | 2, 4, 5 |
| Workshop 3  (Play with AI Imagery) | Analyze a picture | Claude & ChatGPT | Describe the characteristics of the audience in the photo in 150 words.  Describe the characteristics of the audience in the photo in 150 words. Use social constructivism as a lens.  Describe the characteristics of the person who owns the bookshelf in the photo. | Concrete experience | 1, 3 |
| Workshop 3  (Play with AI Imagery) | Analyze a picture | Claude & ChatGPT | Describe the characteristics of the audience in the photo in 150 words.  Describe the characteristics of the audience in the photo in 150 words. Use social constructivism as a lens.  Describe the characteristics of the person who owns the bookshelf in the photo. | Active Experimentation | 1, 3, 5 |
| Workshop 3  (Play with AI Imagery) | Analyze a picture | Claude & ChatGPT | Can I apply this to my teaching? If so, how? | Reflective Observation and  Abstract conceptualization | 2, 4, 5 |
| Workshop 3  (Play with AI Imagery) | Analyze a picture | Claude & ChatGPT | Potential challenges?  Ethical implications?   - What stood out to you during the experience? - How did this experience differ from your expectations? - What role did your assumptions and beliefs play as you approached this experience? - What potential challenges do you foresee? - What are the ethical implications? | Reflective Observation and  Abstract conceptualization | 2, 4, 5 |
| Workshop 4  (Role Plays) | Doctor-patient encounter role play | ChatGPT and Claude | You are 35 year old woman with a history of PCOD, hypertension and obesity. I am a medical student who is trying to assess your condition. Simulate a role play in a conversational style. Reveal symptoms that you are think you are having. Include pauses, expressions and movements that a patient might make. Wait for me to prompt you with the questioning. This should feel like a doctor-patient encounter.  Please provide feedback on how the responses were.  Evaluate the questions asked by the medical student in terms how effective the questions were in eliciting the responses from the patient. | Concrete experience | 1, 3 |
| Workshop 4  (Role Plays) | Doctor-patient encounter role play | ChatGPT and Claude | You are a <enter details> year old with a history of a <enter details>. I am a <enter details> student who is trying to assess your condition. Simulate a role play in a conversational style. Reveal symptoms that you are think you are having. Include pauses, expressions and movements that a patient might make. Wait for me to prompt you with the questioning. This should feel like a doctor-patient encounter.  Please provide feedback on how the responses were.  Evaluate the questions asked by the medical student in terms how effective the questions were in eliciting the responses from the patient. | Active Experimentation | 1, 3, 5 |
| Workshop 4  (Role Plays) | Doctor-patient encounter role play | ChatGPT and Claude | Can I apply this to my teaching? If so, how? | Reflective Observation and  Abstract conceptualization | 2, 4, 5 |
| Workshop 4  (Role Plays) | Doctor-patient encounter role play | ChatGPT and Claude | Potential challenges?  Ethical implications?   - What stood out to you during the experience? - How did this experience differ from your expectations? - What role did your assumptions and beliefs play as you approached this experience? - What potential challenges do you foresee? - What are the ethical implications? | Reflective Observation and  Abstract conceptualization | 2, 4, 5 |
| Workshop 5  (Active  experimentation) | Active experimentation | ChatGPT and Claude | Use GenAI to brainstorm/plan suggested scenarios or use your own  You have been invited to:   - Chair a committee (to review a student?) - Lead a taskforce (need to plan for the inaugural meeting?) - Teach a new class (or revise/revamp material for a course as per new guidelines provided?) | Active Experimentation | 1, 3, 5 |
| Workshop 5  (Active experimentation) | Active experimentation | ChatGPT and Claude | - Describe your scenario briefly. - What went well? - Challenges? | Reflective Observation and  Abstract conceptualization | 2, 4, 5 |
