# Supplementary material for: Design and Evaluation of a Faculty Development Workshop Series on Integrating Generative Artificial Intelligence in Medical Education: Mixed Methods Pilot Study
Source: JMIR Med Educ. 2026 May 6;12:e89815. doi: 10.2196/89815 (PMC13148327; doi:10.2196/89815)
Supplement: Multimedia Appendix 2 [file mededu-v12-e89815-s002.pdf]

Weill Cornell Medicine-Qatar

Institutional Review Board
Approval Date: July 11, 2024
Expiration Date: July 10, 2025
APPROVED

Pre intervention survey instrument

Survey: To protect your confidentiality, please create a unique identifier known only to you. To create this unique code, please use the first three letters of your mother's first name followed by your mother's birth year. Thus, for example, if your mother's name was Sarah and she was born in 1958, the code would be Sar1958. The unique identifier will allow us to match the post-intervention survey responses and the retrospective, pre-intervention responses when we analyze the data.

Please enter your unique identifier: _____ ________ (e.g., Sar1958, see paragraph above)

Demographic information

1. What is your age?
   a. Below 30
   b. 30 - 39 years
   c. 40 - 49 years
   d. 50 - 59 years
   e. 60 +
2. How many years have you been working in Weill Cornell Medicine-Qatar?
   a. Less than a year
   b. 1 to 3 years
   c. 4 to 6 years
   d. 7 to 10 years
   e. More than 10 years
3. Please indicate years of experience
   a. Junior faculty (< 5 years of experience)
   b. Mid-career faculty (5 - 10 years of experience)
   c. Senior faculty (10+ years of experience)
4. How long have you been with your current position?
   a. Less than a year
   b. 1 to 3 years
   c. 4 to 6 years
   d. 7 to 10 years
   e. More than 10 years
5. What is your gender?
   a. Male
   b. Female
   c. Prefer not to disclose

Generative AI usage information

6. How would you rate your understanding of generative artificial intelligence (GenAI) technologies such as ChatGPT (OpenAI), Bing / CoPilot (Microsoft), Gemini (Google), Dall-E2, Claude, Scribe etc.? Please select the most appropriate option below:

a. No knowledge (*I am not familiar with GenAI technologies*)
b. Basic understanding (*I know what GenAI is but have not used it*)
c. Moderate knowledge (*I understand the basics of GenAI and have experimented with it)*
    i. Please provide examples of GenAI experimentation (open text response)
d. Advanced knowledge (*I am well-versed in GenAI technologies and regularly incorporate them into my curriculum*)
    i. Please provide examples of GenAI use (open text response)
e. Expert level (*I have extensive knowledge of GenAI technologies, contribute to scholarly work, and consistently use these technologies to enhance teaching and learning outcomes*)
    i. Please provide examples of GenAI use (open text response)

Directions: For the following sections please indicate your level of agreement with each of these statements regarding medical educator leadership.

1. Strongly disagree
2. Disagree
3. Neither agree nor disagree
4. Agree
5. Strongly agree

For the purpose of this survey, technology refers to GenAI tools such as ChatGPT (OpenAI), Bing / CoPilot (Microsoft), Gemini (Google), Dall-E2, Claude, Scribe etc.

Please answer all the questions.

		Strongly disagree	Disagree	Neither agree or disagree	Agree	Strongly agree
	**TK (Technology Knowledge)**					
7	I know how to solve my own technical problems.					
8	I can learn technology easily.					
9	I keep up with important new technologies.					
10	I frequently play around the technology.					
11	I know about a lot of different technologies.					
12	I have the technical skills I need to use technology.					

PK (Pedagogical Knowledge)

13 I know different ways to assess student performance in a classroom.
14 I can adapt my teaching based-upon what students currently understand or do not understand.
15 I can adapt my teaching style to different learners.
16 I can assess student learning in multiple ways.
17 I can use a wide range of teaching approaches in a classroom setting.
18 I am familiar with common student understandings and misconceptions.
19 I know how to organize and maintain classroom management.

PCK (Pedagogical Content Knowledge)

20a. I can select effective teaching approaches to guide student thinking and learning in medical education.
20b. Name the subject/s you teach (open text response)

TCK (Technological Content Knowledge)

21a I know about technologies that I can use for understanding and teaching content in medical education
21b. Name the subject/s you teach (open text response)

TPK (Technological Pedagogical Knowledge)

22 I can choose technologies that enhance the teaching approaches for a lesson.
23 I can choose technologies that enhance students' learning for a lesson.
24 My teacher education program has caused me to think more deeply about how technology could influence the teaching approaches I use in my classroom.

- I did not attend a teacher education program

25 I am thinking critically about how to use technology in my classroom/teaching sessions.
26 I can adapt the use of the technologies that I am learning about to different teaching activities.
27 I can select technologies to use in my classroom that enhance what I teach, how I teach and what students learn.
28 I can use strategies that combine content, technologies, and teaching approaches that I learned about in my coursework in my classroom.
- I did not attend a teacher education program

29 I can provide leadership in helping others to coordinate the use of content, technologies, and teaching approaches at my institution.
30 I can choose technologies that enhance the content for a lesson.

TPACK (Technology Pedagogy and Content Knowledge)

31a. I can teach lessons that appropriately combine medical education content, technologies, and teaching approaches.

31b. Name the subject/s you teach (open text response)

Institutional Review Board
Approval Date: July 11, 2024
Expiration Date: July 10, 2025
APPROVED

Post intervention survey instrument

Survey: To protect your confidentiality, please create a unique identifier known only to you. To create this unique code, please use the first three letters of your mother's first name followed by your mother's birth year. Thus, for example, if your mother's name was Sarah and she was born in 1958, the code would be Sar1958. The unique identifier will allow us to match the post-intervention survey responses and the retrospective, pre-intervention responses when we analyze the data.

Please enter your unique identifier: _____ ________ (e.g., Sar1958, see paragraph above)

Generative AI usage information

6. How would you rate your understanding of generative artificial intelligence (GenAI) technologies such as ChatGPT (OpenAI), Bing / CoPilot (Microsoft), Gemini (Google), Dall-E2, Claude, Scribe etc.? Please select the most appropriate option below:

    a. No knowledge (*I am not familiar with GenAI technologies*)
    b. Basic understanding (*I know what GenAI is but have not used it*)
    c. Moderate knowledge (*I understand the basics of GenAI and have experimented with it)*
        i. Please provide examples of GenAI experimentation (open text response)
    d. Advanced knowledge (*I am well-versed in GenAI technologies and regularly incorporate them into my curriculum*)
        i. Please provide examples of GenAI use (open text response)
    e. Expert level (*I have extensive knowledge of GenAI technologies, contribute to scholarly work, and consistently use these technologies to enhance teaching and learning outcomes*)
        i. Please provide examples of GenAI use (open text response)

Directions: For the following sections please indicate your level of agreement with each of these statements regarding medical educator leadership.

1. Strongly disagree
2. Disagree
3. Neither agree nor disagree
4. Agree
5. Strongly agree

For the purpose of this survey, technology refers to GenAI tools such as ChatGPT (OpenAI), Bing / CoPilot (Microsoft), Gemini (Google), Dall-E2, Claude, Scribe etc.

Please answer all the questions.

		Strongly disagree	Disagree	Neither agree or disagree	Agree	Strongly agree
	**TK (Technology Knowledge)**					
7	I know how to solve my own technical problems.					
8	I can learn technology easily.					
9	I keep up with important new technologies.					
10	I frequently play around the technology.					
11	I know about a lot of different technologies.					
12	I have the technical skills I need to use technology.					
	**PK (Pedagogical Knowledge)**					
13	I know different ways to assess student performance in a classroom.					
14	I can adapt my teaching based-upon what students currently understand or do not understand.					
15	I can adapt my teaching style to different learners.					
16	I can assess student learning in multiple ways.					
17	I can use a wide range of teaching approaches in a classroom setting.					
18	I am familiar with common student understandings and misconceptions.					
19	I know how to organize and maintain classroom management.					
	**PCK (Pedagogical Content Knowledge)**					
20a.	I can select effective teaching approaches to guide student thinking and learning in medical education.					
20b.	Name the subject/s you teach (open text response)					

TCK (Technological Content Knowledge)

21a. I know about technologies that I can use for understanding and teaching content in medical education

21b. Name the subject/s you teach (open text response)

TPK (Technological Pedagogical Knowledge)

22 I can choose technologies that enhance the teaching approaches for a lesson.
23 I can choose technologies that enhance students' learning for a lesson.
24 My teacher education program has caused me to think more deeply about how technology could influence the teaching approaches I use in my classroom.
- I did not attend a teacher education program

25 I am thinking critically about how to use technology in my classroom/teaching sessions.
26 I can adapt the use of the technologies that I am learning about to different teaching activities.
27 I can select technologies to use in my classroom that enhance what I teach, how I teach and what students learn.
28 I can use strategies that combine content, technologies, and teaching approaches that I learned about in my coursework in my classroom.
- I did not attend a teacher education program

29 I can provide leadership in helping others to coordinate the use of content, technologies, and teaching approaches at my institution.
30 I can choose technologies that enhance the content for a lesson.

**TPACK (Technology Pedagogy and Content Knowledge)**

31a. I can teach lessons that appropriately combine medical education content, technologies, and teaching approaches.

31b. Name the subject/s you teach (open text response)

Workshop experiences and perspectives

Please rate the various aspects of the PDGenAI-P workshops.

32a. How would you rate the content of the PDGenAI-P workshops?
1. Very poor
2. Below average
3. Average
4. Above average
5. Excellent

32b. Please elaborate on your rating. (Open text response)

33a. How would you rate the organization of the PDGenAI-P workshops?
1. Very poor
2. Below average
3. Average
4. Above average
5. Excellent

33b. Please elaborate on your rating. (Open text response)

34a. How would you rate the relevance of the PDGenAI-P workshops?
1. Very poor
2. Below average
3. Average
4. Above average
5. Excellent

34b. Please elaborate on your rating. (Open text response)

35a. How would you rate the overall quality of the PDGenAI-P workshops?
1. Very poor
2. Below average
3. Average

4. Above average
5. Excellent

35b. Please elaborate on your rating. (Open text response)

36. What were the the key learnings from this workshop series? (Open text response)

37. How did this professional development (PDGenAI-P) relate to your teaching, and in what way(s) has it caused you to review your teaching approaches? (Open text response)

38. Did the professional development (PDGenAI-P) meet your expectations? Please explain in detail and include any benefits / drawbacks / limitations / barriers that you experienced. (Open text response)

Post intervention follow up survey instrument

Thank you for participating in the Professional Development in Generative Artificial Intelligence for Pedagogy (PDGenAI-P) workshop series earlier this month. This is part of a research study on the use of generative artificial intelligence (GenAI) in medical education pedagogy.

We invite you to share your PDGenAI-P insights and experiences through this survey. Your feedback will be valuable for refining and enhancing future workshops as well as contribute to the broader understanding of generative AI's impact on educational practices.

Workshop impact: Knowledge and confidence
1. What were the the key learnings from this workshop? (Open text response)

2a. How confident do you feel about applying the knowledge and skills gained from PDGenAI-P in your pedagogical practice?
  a) Not at all confident
     (I do not feel prepared to apply the concepts/skills in my teaching practice)
  b) Slightly confident
     (I feel somewhat prepared but would need more guidance/practice before applying these concepts/skills in my pedagogical practice)
  c) Moderately confident
     (I feel somewhat prepared and might try to apply some of the concepts/skills in my pedagogical practice)
  d) Confident
     (I feel prepared and confident in my ability to apply most concepts/skills learned in my pedagogical practice)
  e) Very confident
     (I feel fully prepared and very confident in my ability to seamlessly integrate all concepts/skills learned in my pedagogical practice)

2b. Please share what factors contribute to your level of confidence? (Open text response)

3a. To what extent have your perspectives on teaching and learning with generative AI changed because of attending PDGenAI-P workshops?
  a) No change at all
     (*My perspectives have not changed after attending the workshops. I maintain the same views as I did before*)
  b) Slight change
     (There has been a minor shift in my perspectives. I see a few things differently but largely hold the same views)
  c) Moderate change
     (*I have gained new insights that have changed my views to some extent)*
  d) Considerable change
     (I have adopted many new views and approaches)
  e) Complete transformation

Institutional Review Board
Approval Date: July 11, 2024
Expiration Date: July 10, 2025
APPROVED

(*The workshops have changed my approach and understanding of generative AI in teaching and learning)*

3b. Please explain any changes in your perspectives or approach to teaching and learning. (Open text response)

Intended implementation strategies and good practices
4a. Which of the following strategies from PDGenAI-P do you plan to implement in your pedagogical practice? Select all that apply.

a) Use generative AI to create case scenarios to facilitate project-based learning
b) Use generative AI to create a lesson plan, interactive activities and a rubric using a backward design model
c) Use generative AI to visualize learning material
d) Use generative AI to create various types of assessments (MCQ’s USMLE style, formative etc.) based on the lesson plan and rubric
e) Use generative AI to adapt learning material to create personalized learning experiences
f) Critically analyze content generated by AI
g) Define responsible use of generative AI in your settings
h) Initiate discussions of responsible and ethical use of generative AI in your classroom
i) Use generative AI for managing administrative tasks
j) Other: Please specify (Open text response)

4b. Please describe how you plan to implement these strategies within your pedagogical practice. (Open text response)

5a. How concerned are you about encountering obstacles in applying what you've learned from PDGenAI-P?

a) Very concerned
(I am highly concerned about several major obstacles and feel I need additional support to apply my learning)
b) Fairly concerned
(I foresee several obstacles and will need to seek solutions to overcome them)
c) Moderately concerned
(I am worried about potential obstacles and am considering strategies to address them)
d) Mildly concerned
(I have slight concerns that there might be minor obstacles, but I feel well-equipped to manage them)
e) Not concerned at all
(I am completely confident in my ability to apply the learning and resolve any obstacles that I might encounter)

5b. Please describe any anticipated obstacles and your strategies for addressing them. (Open text response)

6. What type of additional support or resources do you feel you need to effectively apply your PDGenAI-P learnings? (Select all that apply)

Weill Cornell Medicine-Qatar
Institutional Review Board
Approval Date: July 11, 2024
Expiration Date: July 10, 2025
APPROVED

a) Step-by-step instructions for integrating PDGenAI-P strategies into teaching practices
b) Opportunities to discuss specific challenges and questions with generative AI experts
c) Forums for exchanging ideas and experiences with other educators applying generative AI
d) Additional workshops or training sessions to deepen understanding and address emerging challenges
e) A customized collection of online guides and resources for further exploration and application
f) Guidance on navigating institutional policies and obtaining support for innovative teaching approaches
g) I do not require any additional support.
h) Other support: Please specify (Open text response)

7. How effective do you believe generative AI can be in enhancing medical education pedagogical practices?

a) Not effective at all
(Generative AI offers no benefits and may not enhance pedagogy in medical education in any meaningful way)
b) Slightly effective
(Generative AI may offer minor benefits, but its overall impact on enhancing pedagogical practices in medical education is limited)
c) Moderately effective
(Generative AI can provide some enhancements in medical education pedagogical practices)
d) Effective
(Generative AI has the potential to promote development and innovation in medical education pedagogical practices)
e) Very effective
(I am convinced that generative AI will transform medical education pedagogical practices)

8. Based on your experience, what are some good practices for using generative AI in medical education pedagogical practices? (Open text response)

9. Do you have any additional comments, suggestions, or feedback regarding the PDGenAI-P workshops? (Open text response)
